# Medical applications and prospects of polylactic acid materials

**DOI:** 10.1016/j.isci.2024.111512

**Published:** 2024-12-01

**Authors:** Zhenqi Yang, Guoyong Yin, Shuyang Sun, Ping Xu

**Affiliations:** 1 State Key Laboratory of Microbial Metabolism and School of Life Sciences and Biotechnology, Shanghai Jiao Tong University, Shanghai 200240, P.R. China; 2 Department of Orthopedics, The First Affiliated Hospital of Nanjing Medical University, Nanjing Medical University, Nanjing, Jiangsu 210029, P.R. China; 3 Asia Pacific Graduate Institute of Shanghai Jiao Tong University, Campus for Research Excellence and Technological Enterprise (CREATE), 1 CREATE Way, Singapore 138602, Singapore

**Keywords:** Health sciences, Biomaterials, Biomedical materials

## Abstract

Polylactic acid (PLA) is a biodegradable and bio-based polymer that has gained significant attention as an environmentally friendly alternative to traditional petroleum-based plastics. In clinical treatment, biocompatible and non-toxic PLA materials enhance safety and reduce tissue reactions, while the biodegradability allows it to breakdown over time naturally, avoiding a second surgery. With the emergence of nanotechnology and three-dimensional (3D) printing, medical utilized-PLA has been produced with more structural and biological properties at both micro and macro scales for clinical therapy. This review summarizes current applications of the PLA-based biomaterials in drug delivery systems, orthopedic treatment, tissue regenerative engineering, and surgery and medical devices, providing viewpoints regarding the prospective medical utilization.

## Graphical abstract

**Figure:**
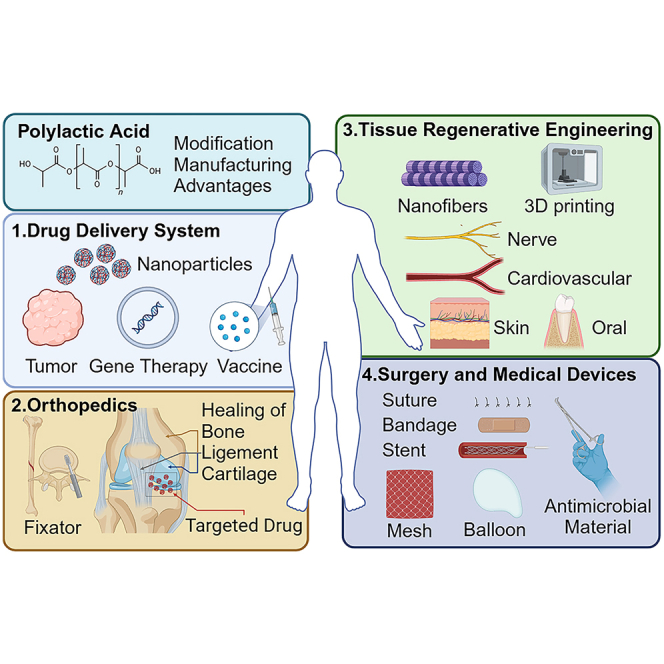

## Introduction to polylactic acid

Polylactic acid (PLA) stands one of the most studied and applied biodegradable aliphatic polyesters in the biomedical field. Produced from fermentation of sustainable sources like corn syrup, potatoes, and sugar cane, PLA is then synthesized by polymerization of lactic acid.^1^ At the end of product life, PLA is degraded hydrolytically into lactic acid, a common metabolic product, and thus considered as biocompatible and environmentally friendly.^2^^,^^3^^,^^4^ PLA has demonstrated the significant potential, either as a substitute for traditional petrochemical-based polymers in industry or as a prominent biomaterial for various medical applications.^5^

### Synthesis of PLA

Lactic acid is the monomer of PLA, and due to its asymmetric carbon atoms, there are two spatial isomers: the dextrorotatory form called r or d-lactic acid and the levorotatory form called s or l-lactic acid.^6^ Since only l-lactic acid exits in mammalian metabolism, d-lactic acid or a mixture of d- and l-lactic acid is generally not advisable in the food, drink, and pharmaceutical industries to avoid metabolic issues.^7^^,^^8^^,^^9^

The manufacturing process of PLA mainly consists of two steps: the preparation of high-purity lactic acid monomers and the synthesis of high molecular weight PLA.^10^ There are two approaches for lactic acid production: chemical synthesis and microbial fermentation.^11^ The primary method for chemical synthesis is through the addition reaction of acetic aldehyde and hydrogen cyanide, followed by hydrolysis to obtain lactic acid. The chemical synthesis approach has problems such as limited production capacity, high costs, and production of only racemic lactic acid.^12^ Therefore, most of lactic acid worldwide is produced through microbial fermentation.^13^^,^^14^^,^^15^ Lactic acid bacterial fermentation can be divided into homofermentative fermentation, where glucose is mainly converted by lactic acid bacteria into lactic acid, counting for more than 90% of the products, and heterofermentative fermentation, where glucose is converted by lactic acid bacteria into various products including lactic acid, ethanol, acetic acid, etc.^16^ Currently, industrial companies commonly use engineered strains such as recombinant *Escherichia coli* to efficiently produce lactic acid monomers by adding neutralizing agents like calcium hydroxide during fermentation.^17^ This method yields over 140 g per liter, which achieves high fermentation intensity and produces only l-lactic acid or d-lactic acid.^18^ Additionally, acid-resistant yeast or *Lactobacillus* can produce lactic acid in non-neutralizing fermentation systems, though the lactic acid yield is relatively low.^19^^,^^20^

The downstream purification process includes the removal of bacterial cells, separation of by-products, and the refining and purification of lactic acid monomers.^21^ Currently, the widely used approach for the separation and purification of lactic acid monomers is the calcium lactate crystallization-acid hydrolysis process, which provides high yield and purity. Due to the technical maturity, operational simplicity, and high efficiency, traditional neutralization precipitation methods are still used in industrial production. Meanwhile, the development of emulsion liquid membrane extraction, reactive distillation, and advanced membrane separation techniques have shown great potential for purifying lactic acid in an energy-efficient manner.^22^^,^^23^

After extraction and purification, the lactic acid monomers are polymerized through a polycondensation reaction to form PLA. There are three primary methods for massive PLA production: direct lactic acid polycondensation, lactic acid azeotropic dehydrative condensation, and ring-opening polymerization.^24^^,^^25^ The direct polycondensation involves the continuous dehydration of lactic acid to form oligomers and then PLA.^26^ However, water is slowly diffused in the viscous polymer melt during processing. The residual water in the PLA melt affects the efficiency of the polycondensation reaction and limits the molecular weight and performance of PLA. Therefore, azeotropic dehydrative condensation is developed for high molecular weight PLA, using a high-boiling-point organic solvent to achieve dehydration. The ring-opening polymerization involves the dehydration and polycondensation of lactic acid to form oligomers under high-temperature vacuum conditions, eliminating water.^27^ Subsequently, these oligomers undergo depolymerization to produce lactide (cyclic dimer of lactic acid) under reduced pressure. Finally, purified lactide, separated from residual water and lactic acid, undergoes ring-opening polymerization reaction to form PLA. By using various catalysts, the ring-opening polymerization method can achieve both high molecular weight and high optical purity and has been the most used method for large-scale production of PLA.^28^^,^^29^

Furthermore, the strategies for synthesizing PLA are constantly evolving.^30^^,^^31^^,^^32^^,^^33^^,^^34^ In addition to optimizing the process for ring-opening polymerization and developing new catalysts, reports have indicated the successful construction of PLA metabolic pathways in chassis cells such as *E. coli*,^35^ *Yarrowia lipolytica*,^36^ and *Saccharomyces cerevisiae*,^37^ using glucose as a substrate for the biosynthesis of PLA homopolymers.^38^ In the biological polymerization process, the key enzyme propionyl-CoA transferase is responsible for converting lactic acid monomers into lactyl-CoA, which is further polymerized into PLA under the catalytic action of polyhydroxyalkanoate synthase. Compared to chemical processes, the biological polymerization of PLA faces challenges such as low polymer content within cells and low molecular weight of the polymers, directly impacting the production costs and material properties of PLA.^35^ Currently at the forefront, synthetic biology has also proposed the utilization of cyanobacteria as host cells for synthesizing PLA directly from carbon dioxide. By employing a combination of metabolic engineering and high-density cultivation on a light-driven cyanobacteria platform, one-step biosynthesis of PLA using carbon dioxide achieved a PLA concentration as high as 108.0 mg/L.^39^ This “negative carbon” production technology can not only address plastic pollution but also achieve carbon capture, offering multiple benefits on society, economy, and the environment.^40^

### Properties of PLA materials

Properties of PLA depend on its component isomers, molecular weight, annealing time, and processing temperature.^41^ Component isomers can affect the crystallinity, melting temperature, and glass transition temperature.^42^ PLA with poly l-lactic acid content higher than 90% tends to be crystalline, while a lower proportion often leads to amorphous PLA with reduced melting temperature and glass transition temperature.^43^ Molecular weight primarily affects the degradation, mechanical strength, and solubility of polymers. PLA materials with a high molecular weight take approximately 2–8 years for complete resorption.^44^ The PLA used in implants, such as orthopedic fixation screws, has a higher molecular weight, providing mechanical strength for a period, allowing the bone fracture to heal properly. In contrast, PLA materials used in drug delivery systems often have lower molecular weights, allowing for relatively rapid degradation and reduced drug accumulation in tissues.^44^

### Modification of PLA materials

Copolymerization of lactic acid and different monomers together may control the degradation time of PLA materials or enhance their hydrophilicity, flexibility, and ductility.^45^^,^^46^ For example, copolymerization of lactic acid and glycolic acid forms poly(lactic-co-glycolic) acid (PLGA), which can shorten the degradation time of PLA from 6 months to 2 months.^47^ Since the degradation time is negatively correlated with the content of polyglycolide, the biocompatible PLGA can be used in drug delivery systems with controlled medicine release.^46^^,^^48^^,^^49^^,^^50^^,^^51^ In addition, hydrogels formed by copolymerization of lactide and polyethylene glycol (PEG) exhibit good biocompatibility, adjustable degradation rates, and enhanced flexibility.^48^^,^^52^ These hydrogels can absorb large amounts of water, forming soft gel networks with excellent plasticity, and then be used in drug delivery systems and as cell carrier scaffolds for tissue regeneration engineering.^53^^,^^54^^,^^55^ Moreover, the PLA-polycaprolactone copolymer is a semi-crystalline, biodegradable thermoplastic with good flexibility and a low melting point (∼60°C).^56^^,^^57^ Compared with PLA, this copolymer demonstrates enhanced flexibility and ductility, reduced brittleness, and improved mechanical properties, making it an ideal material for biodegradable implants and three-dimensional (3D) printing.^58^^,^^59^

Blending PLA with other polymers through melting or solution mixing can significantly change its properties,^60^^,^^61^ offering a cost advantage over copolymerization.^61^^,^^62^ Blending with heat-resistant polymers can be used for heat-resistant flame-retardant materials, while blending with flexible polymers can improve PLA’s toughness and elasticity for 3D printing applications.^63^ However, the application of PLA blended materials in the medical field is relatively limited.^64^

PLA materials can also be modified by adding fillers or plasticizers.^65^ Since PLA is hydrophobic and lacks inherent bioactivity, appropriate fillers can promote cell migration, extracellular matrix deposition, and vascularization.^50^^,^^55^ Calcium-based inorganic fillers, such as calcium phosphate and calcium silicate, have been shown to promote the migration and mineral deposition of mesenchymal stem cells, making them widely applied in PLA modification for bone tissue regeneration.^66^^,^^67^

### Advanced manufacturing for PLA

Various processing techniques have been developed to meet the thermal and mechanical demands of PLA materials, which include but not limited to drying and extrusion,^68^ injection molding,^69^ injection stretch blow molding,^3^ and casting (film and sheet).^70^ PLA can also be transformed into films, fibers, particulates, and porous structures of various shapes and sizes through techniques like solvent blending or melt blending.^62^ These processes have largely broadened the applications of PLA materials.^70^^,^^71^

PLA can be engineered into nanomaterials, unlocking exciting possibilities for various applications.^72^ Advanced techniques like nanoparticle fabrication, electrospinning, and nanocomposite synthesis are employed in producing PLA nanomaterials.^73^ These methods enable precise control over the size, shape, and composition of the nanoparticles or nanofibers. For example, PLA nanoparticles have been employed in drug delivery systems to enhance drug solubility and release profiles.^74^ PLA nanofibers are utilized in applications such as tissue engineering scaffolds, wound dressings, and filtration membranes due to their high surface area-to-volume ratio and favorable mechanical properties.^75^

3D printing is an innovative and forward-looking technology that enables the fabrication of multiscale, biomimetic structures according to specific patient needs and clinical requirements.^76^ In clinical applications, 3D printing technology can be utilized to manufacture personalized medical devices, orthopedic scaffolds, artificial tissues, and biomedical models. In the treatment of bone defects, 3D printing, guided by imaging technologies like magnetic resonance and CT scans, can easily reconstruct the required 3D models. This enables the precise manufacturing of bone grafts that match the defect area accurately.^77^

### Advantages of PLA materials in clinical treatments

Compared with other bio-polymers, PLA is characterized for its biocompatibility, biodegradability, and plasticity in medical applications. Firstly, PLA is not toxic or carcinogenic and it undergoes degradation through non-enzymatic hydrolysis, where its monomer lactic acid is a common metabolic product and eliminated through the tricarboxylic acid cycle.^78^^,^^79^ Secondly, the degradation rate of PLA can be controlled by adjusting factors such as molecular weight, d/l isomer purity, and environment temperature. The degradation time can vary from several hours in drug delivery systems to several months in orthopedic fixation implants, which allows PLA to achieve controlled degradation suitable for various medical treatments and repairs.^44^ Thirdly, PLA can be processed into various forms, including films, fibers, particles, and porous structures.^62^ New manufacturing techniques such as 3D printing technology and nanomaterials further expand its applications in the medical field.

Therefore, PLA materials find a wide range of applications in the clinical treatments.^80^ PLA and PLA blends have been investigated for various drug delivery approaches, encompassing nanosystems, hydrogels, films, and fibrous matrices.^81^ In orthopedic and dental applications, PLA-based materials have found widespread use as fixation devices such as pins and screws in reconstructive surgeries for various fractures.^82^ In tissue regenerative engineering, PLA-based biomaterials bring new developments for bone, ligament and cartilage regeneration.^75^ PLA can also be used in wound management, such as surgical sutures.^83^

## The PLA application in drug delivery system

### Improved drug delivery systems through PLA nanoparticles

The introduction of controlled release drug delivery has brought a significant transformation in the pharmaceutical sector, which provides the advantages of low side effects and specific targeting abilities.^84^^,^^85^ PLA and its copolymers have been extensively investigated in this field because of their remarkable biocompatibility, biodegradability, low immunogenicity, and desirable mechanical properties.^86^ For instance, PLA and PLGA have been approved by FDA for human use in formulations for controlled release drug delivery and as carriers for vaccine antigens.^87^ Carried by PLA nanoparticles, macromolecules such as nucleic acids, peptides, proteins, etc., now can be used in therapeutics, by overcoming the challenges of rapid clearance through kidney or liver, enzyme degradation, cell membrane permeability, barriers between blood and other organs.^88^^,^^89^ Additionally, nanoparticle drug delivery system can enhance the performance of various medications beyond conventional formulations, by reducing the size of compounds and modifying the surface characters.^90^ For instance, nanoparticle drug delivery system can be utilized to encapsulate drugs, leading to (1) improved solubility especially for hydrophobic drugs, (2) protection from degradation, (3) increased absorption through epithelial barriers and extended circulation in the bloodstream, (4) precise targeting of drugs to specific cells, tissues, or organs, and (5) enhanced cellular uptake. A few methods have been employed to produce PLA nanoparticles, such as single or multiple emulsion,^91^^,^^92^ nano precipitation or salting out,^93^ melting based direct compositing method, supercritical fluids technique,^94^ and template/mold based technique.

### Targeted therapy in oncology

Due to the presence of blood-tumor barrier, conventional drugs generally struggle to reach sufficient concentrations at tumor sites, especially for central nervous system tumors that are further blocked by blood-brain barrier.^88^^,^^95^^,^^96^ In oncology, loading usually hydrophobic chemotherapy drugs onto PLA nanoparticles is a promising approach, which can enhance the targeting effect, increase drug concentration at the tumor site, reduce systemic toxicity, and particularly, facilitate penetration through barriers such as the blood-brain barrier.^97^ Additionally, modifications can be made to PLA nanoparticles to change surface chemistry, such as surface charge, influencing the pathways of cellular uptake; or add macromolecules such as antibodies, proteins, nucleic acids, etc., enhancing targeted actions on tumors; or manipulate the chemical composition of nanoparticle themselves, controlling the degradation rate and drug release rate, enhancing drug metabolism kinetics.^98^^,^^99^^,^^100^ For example, a study employed PEG-PLA nanoparticles to deliver peptides for effective tumor targeting and internalization. The F3 peptide, which specifically bound to the overexpressed nucleolin marker on glioma cells, was used to modify the PEG-PLA nanoparticles, resulting in greater accumulation in the glioma regions.^101^

### Delivering polynucleotides and peptides in gene therapy and vaccination

Gene therapy operates on the fundamental idea that polynucleotides delivered into cells can change the expression of a specific protein, leading to therapeutic advantages. This process encompasses the delivery of polynucleotides like DNA, RNA, anti-sense oligonucleotides, and small interfering RNA, either at a specific location or throughout the entire system.^102^ Compared to viral vectors, non-viral vectors are mostly non-immunogenic, cost-effective, safer, and capable of carrying more genetic material. Among them, PLA nanoparticles can serve as non-viral carriers to deliver functional nucleic acids.^103^^,^^104^ It has been reported that biodegradable polymeric nanoparticles through the combination of PLA, PLGA, and polyethylenimine exhibited DNA binding capabilities and performed well *in vitro.*^105^

The therapeutic proteins and peptides have been approved by FDA to cure many diseases, including Alzheimer’s disease, diabetes and melanoma.^106^^,^^107^^,^^108^ The nanoparticle-based systems have significantly enhanced the delivery of antigens for vaccine therapies, which can be custom-designed to resemble cellular components so that they can enter cells through endocytosis and pinocytosis.^109^ Tetanus toxoid, serving as a representative antigen, was integrated into either PLA or PEG-PLA nanoparticles to facilitate nasal vaccine administration, and the properties of these formulations were examined both in laboratory settings and in living organisms.^110^

## The PLA application in orthopedics

### The PLA materials in orthopedic fixation devices

PLA-based materials have found extensive use as fixation devices in orthopedic and dental applications.^82^ Current orthopedic surgical procedures primarily involve the use of autografts, allografts, and metal and plastic implants.^111^ However, metal and plastic implants face various challenges, including low fatigue strength, creep, poor adhesion, and biocompatibility issues with native tissue. Compared to traditional metal fixation materials such as steel or titanium alloys, PLA polymers with modifications to improve mechanical properties and corrosion and creep resistance, can also withstand the loads inside the body.^112^^,^^113^ Moreover, PLA materials have advantages in biodegradability, and the degradation rate can be adjusted by modifications to match the patient’s tissue healing needs. Biodegradability also allows PLA materials to release stress to the affected area over time, facilitating tissue healing. Another significant benefit is the avoidance of a secondary surgical procedure to remove unnecessary hardware. This not only lowers medical expenses but also enables the gradual restoration of tissue function.^82^^,^^114^

PLA polymers have been utilized in orthopedics for the fabrication of biodegradable screws, fixation pins, plates, and suture anchors,^115^ which have been increasingly used in fractures in knee, shoulder, ankle, and foot.^116^^,^^117^ The ligaments in knee and ankle joints are susceptible to tears or injuries because they bear weight and are exposed to high stress. During the surgical for reconstructions, orthopedic screws made by biodegradable polymeric are employed either independently or in conjunction with grafts, depending on the nature of the ligament injury.^118^ Synthetic bioabsorbable screws have been integrated with bioceramic osteoconductive materials such as calcium phosphates and other composites to create biocomposite-based interference screws.^119^

### The PLA based drug delivery system for osteoarthritis

Osteoarthritis is characterized by progressive cartilage degeneration and inflammation, leading to severe joint pain.^120^ Traditional intra-articular drug injection therapies often provide only short-term benefits due to the rapid clearance of drugs from the joint space. The transport of drugs into cartilage to reach cellular targets is also hindered by the dense and negatively charged extracellular matrix of cartilage.^121^^,^^122^ Therefore, developing drug delivery systems capable of crossing the cartilage barrier and achieving long-lasting therapeutic responses has become a research focus. Among these, PLA nanoparticles have garnered significant attention due to their controllable degradation properties and modifiable surfaces, which enable prolonged retention of drugs within the joint and penetration of the extracellular matrix to deliver drugs specifically to diseased chondrocytes. Current research directions include the following aspects. (1) Development of synovial joint-targeted hybrid systems: this involves using a combination of hydrogels, liposomes, and nanoparticle carriers to target the synovial joint and alleviate pain and inflammation.^123^^,^^124^^,^^125^ (2) Drug delivery systems targeting intra-cartilage components: these systems aim to penetrate cartilage by targeting components such as aggrecan, collagen II, and chondrocytes through surface protein or charge modifications, facilitating drug transport through the cartilage.^126^^,^^127^ (3) Steroid-encapsulated polymer microparticles: these microparticles, which have been approved for clinical use, provide prolonged release of steroids to alleviate pain more effectively.^128^^,^^129^

**Figure 1:**
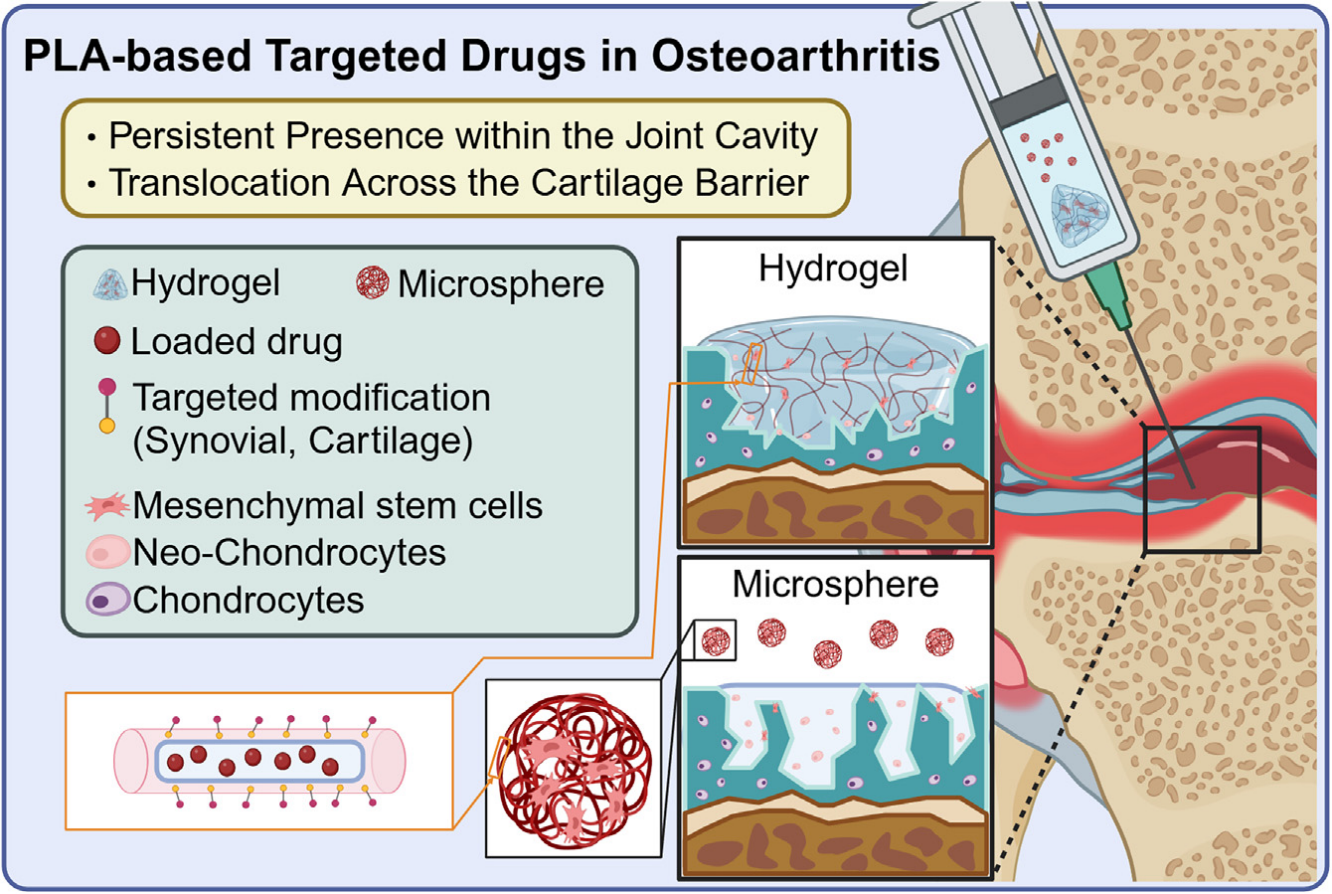
Advantages and strategies of PLA-based targeted drug in osteoarthritis PLA-based targeted drugs in osteoarthritis can persist within the joint cavity for extended periods and effectively translocate across the cartilage barrier due to their controlled degradation properties and modifiable surfaces.^134^ This figure illustrates two common strategies for PLA-based drug delivery: hydrogels and microspheres. Upon injection into the joint cavity, both hydrogels and microspheres form repair zones at cartilage defect sites. Stem cells encapsulated within these delivery systems can differentiate into new chondrocytes under the influence of the drugs, facilitating cartilage repair in osteoarthritis.^133^ Both delivery systems utilize PLA-based nanofibers as fundamental units, capable of loading therapeutic agents and being modified to target synovium or cartilage. Common drugs in these systems include dexamethasone^135^ and kartogenin.^133^

A novel multi-arm cationic nanostructure based on PLA has recently been developed, which possesses 28 covalent drug conjugation sites, allowing it to penetrate the entire thickness of the cartilage at high concentrations. When conjugated with dexamethasone, it achieves prolonged intra-cartilage retention and sustained drug release for up to two weeks.^130^ PLA nanoparticles can also be loaded with various regenerative drugs and combined with structures such as hydrogels and cartilage cell membranes to serve as stem cell expansion vectors.^122^^,^^131^^,^^132^ It has been reported that a novel porous microsphere was made by PLGA and loaded with kartogenin, serving as a stem cell expansion vector. Its advantages include a high cell-carrying capacity (up to 1 × 10^4^ cells/mm^3^) and the ability to effectively protect stem cells, promoting their controlled release in the osteoarthritis microenvironment, and inducing the differentiation of mesenchymal stem cells into chondrocytes (Figure 1).^133^

### The PLA materials in orthopedic tissue regeneration engineering

The bone formation process, known as osteogenesis, can occur through either endochondral ossification or intramembranous ossification pathways.^136^^,^^137^ In addition to being used as fixation devices, PLA is also utilized as a repair and filler material in orthopedics, dentistry, and neurosurgery.^138^ In these fields, PLA can be used for bone defect repairs, providing temporary support and promoting the formation and regeneration of new bone.^139^ Common applications include cranial bone plates in cranioplasty,^140^ bio-substitute materials for mandibular fractures, bioabsorbable bone plates for orbital floor fracture^141^ and filler materials for large bone defects.^142^ Repair and filler materials made by PLA can also carry drugs, controlling the release of drugs during slow degradation to maintain the local concentration. The common drugs loaded are antibiotics that inhibit infection and various growth factors that promote tissue regeneration (Figure 3A).^48^

**Figure 2:**
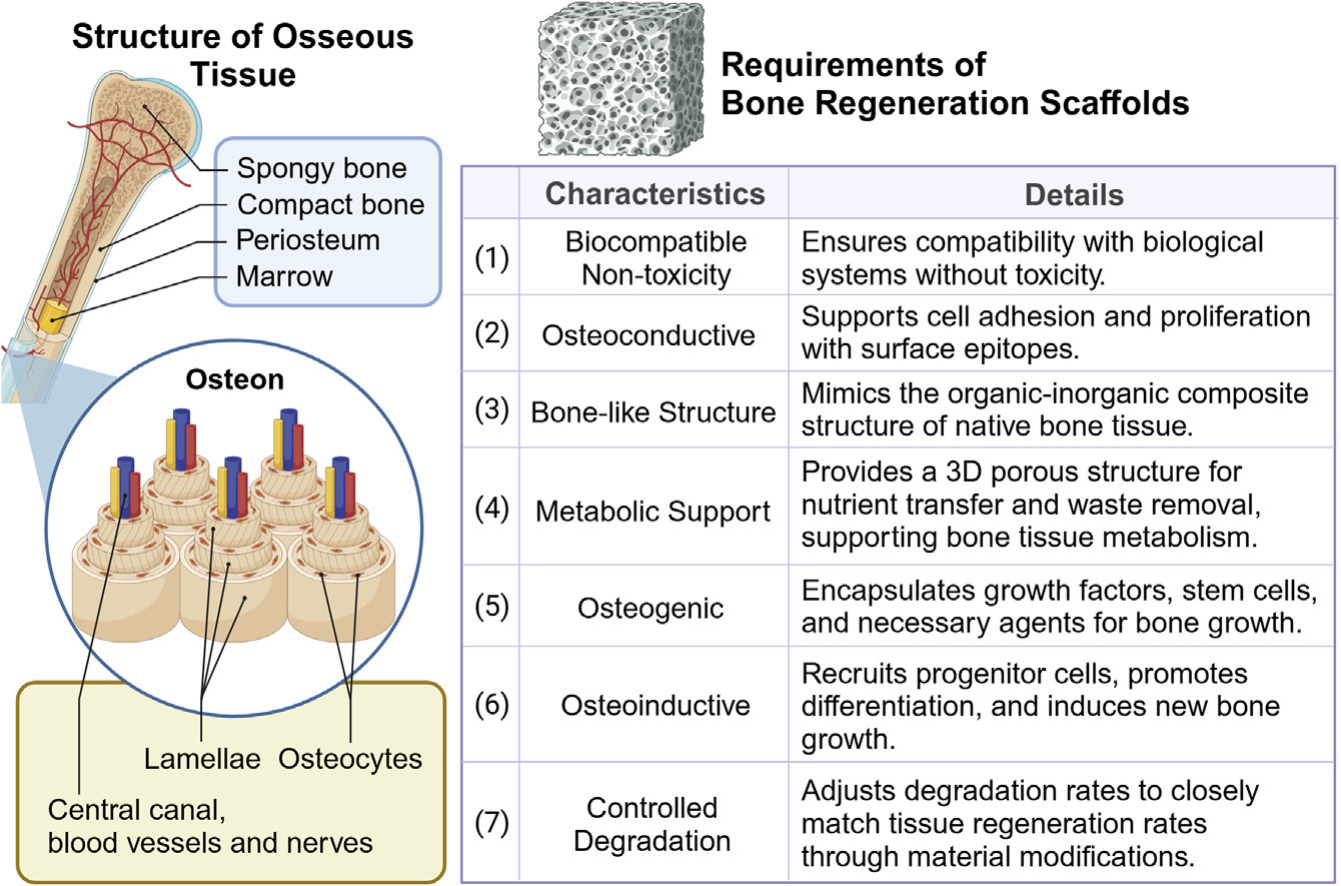
Bone structure and the characteristics required for biomaterial-based bone regeneration scaffolds Bones are essential components of the human skeletal system with a complex anatomical structure. Osteon is the fundamental structural unit of bone, comprising a central canal, concentric bone matrix layers, and osteocytes embedded within.^136^^,^^137^ Osteocytes are responsible for the production of new bone tissue. The central canal contains blood vessels and nerves, ensuring nutrient supply to the bone. The ordered arrangement of osteons imparts strength and stability to the bone. Biomaterial-based scaffolds for bone regeneration are expected possess several beneficial characteristics.^48^^,^^139^

Moreover, researchers have explored more complex scaffold designs and material modifications to better support bone formation and the healing process. The characteristics required for bone tissue regeneration scaffolds are summarized in Figure 2. In orthopedics, the latest technologies involve the use of 3D printing and electrospinning to fabricate composite scaffolds comprising organic and inorganic phases. Polyhydroxyapatite/PLA 3D composite scaffolds have been investigated in bone repair and exhibited improved compatibility, bioactivity, and osteoinductivity, with a reduced likelihood of inflammation.^143^ Furthermore, the incorporation of antibacterial silver nanoparticles, graphene oxide, and selenium nanoparticles made from PLA materials, on the surface or within bone regeneration scaffolds, has been proven to be highly effective for addressing the issues of infection and accelerating bone regeneration after bone tumor treatment.^144^^,^^145^ Also, coordinated growth factors, such as bone morphogenetic proteins, vascular endothelial growth factors, and fibroblast growth factors can be added to the scaffolds for stable and controlled release within the core region of bone regeneration.^146^^,^^147^ A recent study reported a porous bio-composite PLA scaffolds integrated with nuciferine-loaded chitosan hydrogel through 3D printing, which demonstrated uniform pore distribution, sustained nuciferine release, and favorable cytocompatibility with mouse mesenchymal stem cells, leading to enhanced new bone formation.^148^

During ligament injury, the anterior cruciate ligament that lacks vascular tissue is typically difficult to heal and thus biomaterial-based regeneration has been employed.^149^ Multiphasic scaffolds, designed to mimic the process of ligament-cartilage-bone regeneration, have been investigated for osteochondral regeneration.^150^ In a recent report, braided PLGA is used to build multiphasic scaffolds, where the upper phase includes PLGA microspheres to encourage the development of non-calcified cartilage, the middle phase includes a small amount of bioglass added to PLGA microspheres to aid in cartilage tissue calcification, and the lower phase comprises PLGA microspheres with a higher bioglass concentration, promoting bone regeneration.^151^

**Figure 3:**
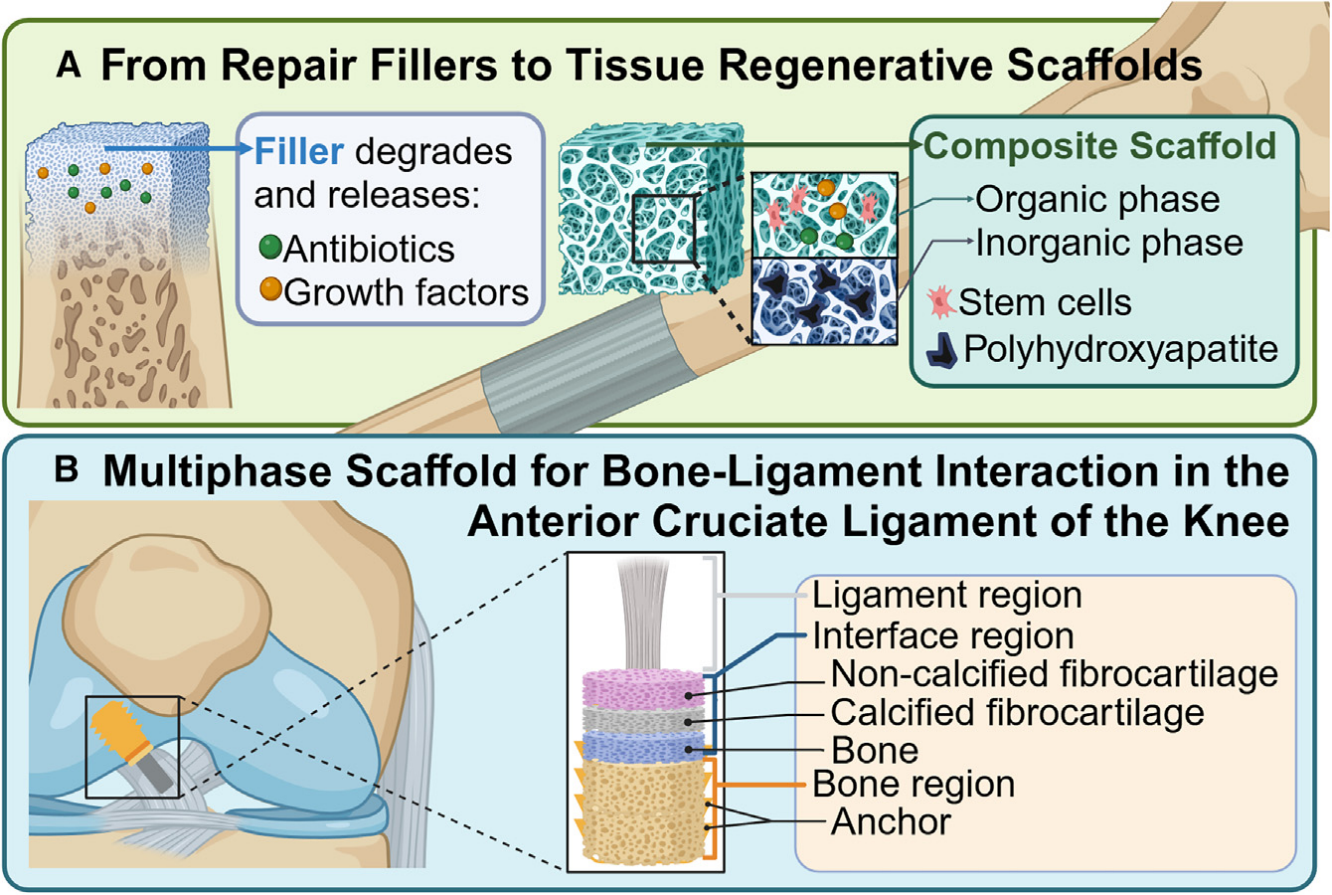
From repair fillers to composite and multiphase tissue regenerative scaffolds (A) In orthopedics, PLA was initially used for manufacturing repair fillers for bone defects.^139^ These fillers are dense in structure and possess high strength, gradually degrading over time within the bone defect area while releasing antibiotics and bone regeneration-promoting growth factors.^48^ As the filler degrades, new bone tissue grows into the defect site. With advancements in stem cell technology and 3D printing, repair fillers have evolved into composite tissue regenerative scaffolds. These scaffolds feature larger pores and are composed of both inorganic and organic phases.^153^ The inorganic phase includes fillers such as hydroxyapatite, which promotes bone mineral deposition.^154^ The organic phase is loaded with stem cells, antibiotics, and growth factors. (B) According to clinical practice, the latest multiphase scaffolds are frequently used for complex defects spanning bone, ligament, and joint tissues.^50^ These scaffolds provide more comprehensive tissue repair and regeneration by replicating the natural transitions between different types of tissues. A multiphase bone-ligament scaffold for treating anterior cruciate ligament injuries. The scaffold includes three regions: the ligament region directly connecting with the ligament repair material, the bone region containing anchor points for fixation and a porous scaffold to promote bone regeneration, and the bone-ligament interface region replicating the gradual transition from fibrocartilage to bone, inducing a mechanical fixation to long-term biological fixation between the implant and the host bone.^151^^,^^155^

In the mid-to-late stages of osteoarthritis, when irreversible cartilage defects occur, drug treatments become very limited. At this point, additional surgical interventions are required. The main clinical treatments currently include microfracture, osteochondral transplantation, and autologous chondrocyte implantation. A recent study reported a novel 3D nanocomposite scaffold made from poly-ε-caprolactone and PLA, incorporating transforming growth factor β 1 (TGF-β1)-loaded chitosan-dextran nanoparticles, and fabricated using the electrospinning method to create a bead-free, semi-aligned nanofiber structure. The scaffold’s advantages include its biomimetic properties, high hydrophilicity, high porosity, and the sustained release of TGF-β1, which collectively enhance the expression of aggrecan and collagen type Ι genes, crucial for cartilage tissue engineering (Figure 3B).^152^

## The PLA application in tissue regenerative engineering

### Tissue regenerative engineering

The concept of tissue regenerative engineering has arisen from the fusion of tissue engineering, advanced materials science, stem cell research, and developmental biology with the aim of regenerating complex tissues that have been damaged.^156^ Advanced materials science offers scaffolds featuring precise geometry and architecture, ensuring sufficient mechanical support and potentially regulating cellular activities by delivering chemical and biochemical materials in both spatial and temporal control.^156^

The PLA-based nanofibers in tissue engineering possess unique characteristics, such as a high surface area-to-volume ratio, high porosity, and excellent mechanical strength.^105^ Additionally, PLA nanoparticles containing growth factors or antimicrobial agents can also be embedded in regenerative scaffolds.^157^ PLA nanoparticles can also be coated on the surface of regenerative scaffolds to improve the compatibility of the scaffold’s surface with tissue materials.^158^ Additionally, the polymer surface can be modified by adding chemically charged end groups (e.g., –OH^–^, –COO^–^, and –NH_3_^+^) to increase protein adsorption, or incorporating peptide segments or synthetically engineered proteins to enhance cell receptor binding and guide cell migration.^159^

### The PLA materials in nervous tissue engineering

**Figure 4:**
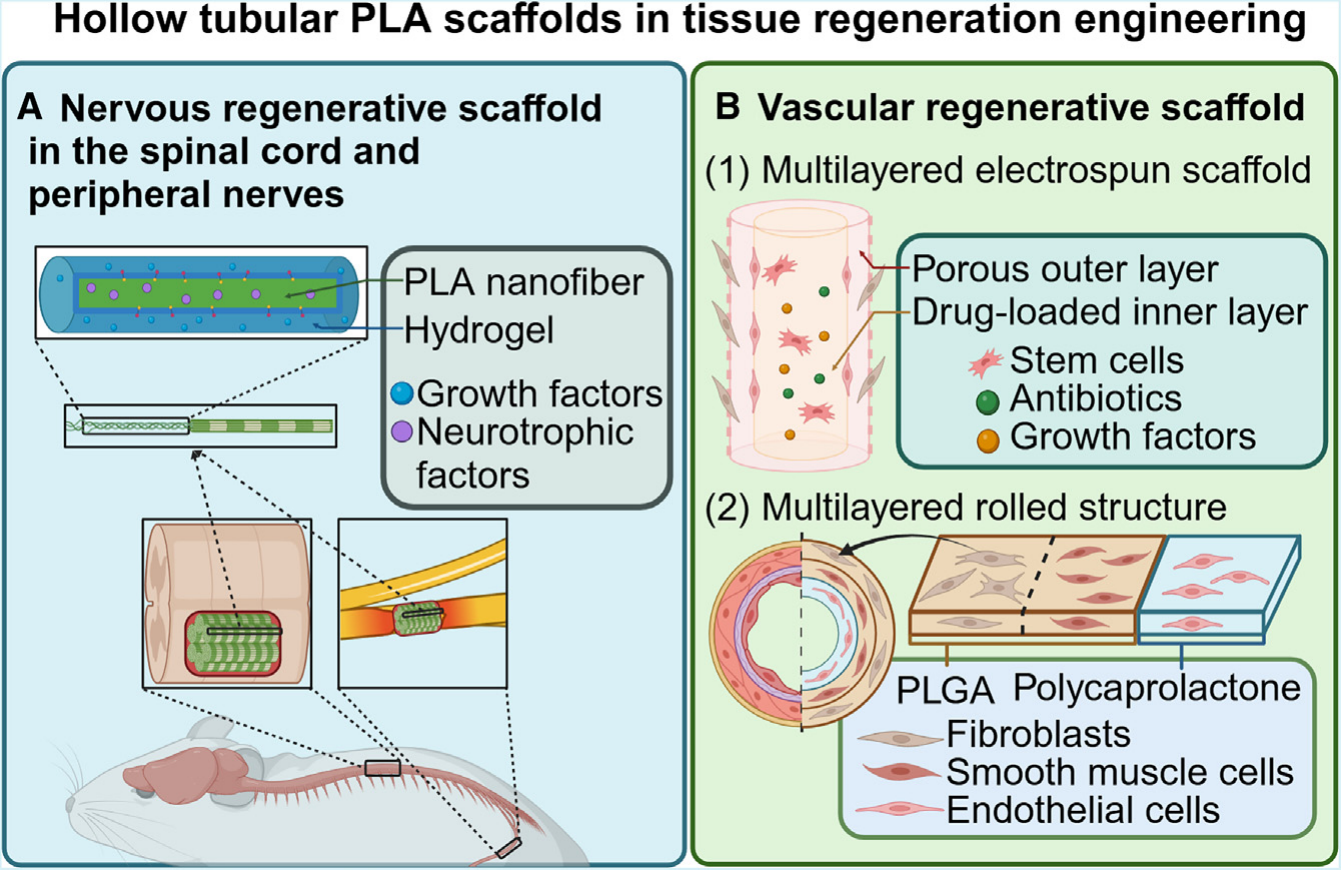
Hollow tubular PLA scaffolds in tissue regeneration engineering In tissue engineering, PLA scaffolds with a hollow tubular scaffold play a significant role in nerve tissue regeneration and cardiovascular tissue regeneration. This scaffold can mimic the morphology of natural tissues, such as nerve conduits and blood vessels, providing an appropriate environment for cell growth, tissue repair, and drug delivery. (A) Nerve regenerative scaffolds are commonly used to repair and regenerate damaged nerves, particularly in spinal cord^164^ and peripheral nerve injuries.^166^ A typical structure consists of a hollow tubular form, composed of PLA nanofibers coated with a hydrogel layer on the surface as the basic unit. This scaffold can carry neurotrophic factors and tissue growth factors that are gradually released over time to promote nerve cell growth and repair. (B) Vascular regenerative scaffolds are primarily used for small-diameter blood vessels and myocardial repair, divided into two common structures. The first is a multilayer tubular electrospun scaffold, featuring a porous outer layer that supports cell growth and tissue repair, facilitating the adhesion of endothelial cells and fibroblasts, and promoting capillary infiltration and endothelial regeneration.^167^ The inner layer is drug-loaded, incorporating stem cells, antibiotics, and tissue regeneration factors.^168^ The second structure is a multilayer rolled structure made from layered PLA composites, loaded with vascular-related cells. After rolling, it forms an arrangement of three types of vascular-related cells (endothelial cells, smooth muscle cells, and fibroblasts) from the inner to outer layers, mimicking the structure of artificial blood vessels. The inner layer degrades slowly to maintain vascular stability, while the outer layer degrades to promote cell growth and angiogenesis.^169^^,^^170^

The effective treatment of severe peripheral nerve injuries remains an unmet clinical challenge, and biomaterials provide a hopeful avenue for stimulating nerve regeneration.^160^^,^^161^^,^^162^ Scaffolds used in neural tissue engineering are typically manufactured in hollow tube-like structures, characterized for biocompatibility and biodegradability. They are designed to be neuro-compatible to support cell growth and the release of neurotrophic factors, thereby promoting regeneration.^163^^,^^164^ Recent study reported that a multi-channel scaffolds made from electrospun poly-l-lactic acid and poly-ε-caprolactone have been developed, in which a suspension of autologous adipose-derived stromal and stem cells was injected during implantation in order to bridge a 10 mm gap in the rat sciatic nerve.^143^ However, while the scaffold was observed to support nerve regeneration during the 4 weeks recovery period, the implanted cells also induced an inflammatory response.^165^ Further research has reported the enhancement of neural repair in rats following spinal cord injury, by integrating PLA nano scaffolds fabricated using electrospinning and hydrogel coatings on the scaffold surfaces. Additionally, regenerative factors like brain-derived neurotrophic factor and stromal cell-derived factor-1α were incorporated into both the scaffolds and coatings, which released over time and promoted neural repair (Figure 4A).^164^

### The PLA materials in cardiovascular tissue engineering

Cardiovascular disease is the most common cause of death globally, prompting a massive need of cardiovascular tissue engineering.^171^ PLA nanofibrous have been used in vascular tissue engineering, especially in small-diameter blood vessels and cardiac muscle repairing.^172^^,^^173^ The model of PLA fibrous vascular scaffolds, like nerve conduit scaffolds, is a tube with a hollow lumen.^73^ Researchers have built a double-layered electrospun scaffold composed of a micro-scale PLA fibrous outer layer and an inner layer made by silk fibroin-gelatin nanofibrous. The outer layer was found to promote the growth and multiplication of mouse fibroblasts, while the inner layer facilitated the adhesion and growth of human umbilical vein endothelial cells. During treatment, there was less inflammatory response and more coinciding between a vascular network forming and the implant denigrating.^174^ Additionally, a self-regulating multilayered polymeric rolled structure composed of polycaprolactone and PLGA has been reported for the fabrication of artificial blood vessels. This tubular structure is formed by the stress-induced rolling of composite polymer membranes and is loaded with three types of vascular cells (endothelial cells, smooth muscle cells, and fibroblasts). After rolling, the structure features an inner layer of polycaprolactone and an outer layer of PLGA, with the three cell types arranged in a manner similar to natural blood vessels (Figure 4B). During degradation, the inner polycaprolactone layer expands outward while the outer PLGA layer contracts inward, effectively maintaining the structural stability of the artificial vessel and facilitating the growth of the loaded cells.^169^

### The PLA materials in skin tissue engineering

Skin is the body’s primary defense against external factors, and a compromised skin barrier can lead to various problems such as infection, hydration issues, and temperature dysregulation.^175^ Nanofibrous mats made by PLA are exceptionally appropriate for promoting skin healing. The mesh structures replicate the natural structure of the dermal bed, aiding in wound protection, moisture retention, protein preservation, and exudate removal.^176^ The large surface area-to-volume ratio of nanofiber mesh structures further promotes cell adhesion and growth, thereby facilitating the healing of large-area wounds.^177^ Also, PLA nanofibrous mats can incorporate drug delivery systems of antibiotics,^178^ silver nanoparticles,^179^ or growth factors^180^ to prevent infection, facilitate wound healing and promote the maturation of skin tissues.

### The PLA materials in oral medicine

In dental, oral, and craniofacial tissue regenerative engineering, including teeth, dental pulp, periodontal tissues, hard and soft tissues of the craniofacial complex, polymeric materials have a broad range of applications as tissue engineering scaffolds, carriers for cell-based therapies, and delivery devices for drugs and biologics.^54^ In oral medicine, PLA materials have been widely applied in the reconstruction and repair of bone tissues, especially in the alveolar bone, sinuses, and temporomandibular joint. The utilization of 3D printing technology to manufacture personalized bi-phasic bone and cartilage scaffolds, followed by seeding the scaffolds with isolated cells and bio-active molecules to promote bone formation, has been demonstrated to achieve favorable clinical outcomes.^181^ Research has shown that in periodontal surgery for the treatment of periodontal intrabony defects, the use of nanohydroxyapatite powder in combination with PLA/PLGA as bone replacement grafts has improved postoperative periodontal parameters in patients.^182^ Additionally, PLGA is efficacious in promoting chondrogenesis by facilitating the colonization and proliferation of mesenchymal stem cells, and it interacts with chondrocytes and other cells in the temporomandibular joint disc.^183^ In periodontal and peri-implant tissue repair and dental pulp regeneration, regenerative scaffolds are primarily used post-periodontal surgery to prevent oral epithelial and soft tissue infiltration into bone defects, and promote the regeneration of periodontal soft and hard tissues.^184^ For more complex dental pulp tissue regeneration, PLA materials combined with stem cell therapy hold significant potential. Recently a study presented an innovative approach for dental pulp regeneration by injecting simvastatin-functionalized GelMA cryogel microspheres loaded with stem cells from human exfoliated deciduous teeth. The system enhances stem cell functions, promotes vascularized pulp-like tissue formation, and demonstrates significant potential for clinical use in endodontic regenerative dentistry.^185^

## The PLA application in surgery and medical devices

### The PLA materials in suturing and surgery

PLA and its copolymers have found extensive applications in surgical sutures and wound management. Because of the biocompatibility and biodegradability of PLA, PLA based suture is well-tolerated by the human body and cause less inflammation or foreign body reactions. Also, PLA breaks down naturally in the body, which is particularly beneficial in surgical sutures, as it eliminates the requirement for a second surgery to remove non-absorbable sutures.^54^ PLA-based sutures can be engineered to have the necessary strength and flexibility for various surgical applications, so that surgeons can choose sutures with specific properties to suit the type of tissue being sutured.^186^ Recent research has reported a new type of antibacterial PLA suture, which uses polyglycolide and polycaprolactone coatings as carriers to load the antibacterial drug ciprofloxacin onto PLA sutures. This drug-loaded antibacterial PLA suture has the dual advantages of being antibacterial and biodegradable.^187^

The PLA materials can also serve as novel bandages and dressings for wound healing. Compared to traditional gauze and other biological dressings such as polyvinylpyrrolidone and sodium alginate fibers, PLA-based biomaterials exhibit more collagen deposition, angiogenesis, and cellular activity, resulting in accelerated wound healing.^188^^,^^189^ This is attributed not only to PLA’s good biocompatibility and biodegradability but also to its moisture management properties, which mimic the extracellular matrix by absorbing and transferring moisture in the wound environment.^190^ Additionally, when PLA materials are combined with antimicrobial agents such as antibiotics, silver, etc., they demonstrate enhanced ability to prevent wound infections.^191^^,^^192^ Recent research reported a novel sandwich-structured PLA-based dressing for hard-to-heal diabetic wounds.^193^ This dressing is composed of an electrospun three-layer structure of PLA-polyvinyl alcohol-PLA. The hydrophilic polyvinyl alcohol inner layer is loaded with metformin hydrochloride, which is slowly released during the wound healing process, promoting the healing of diabetic wounds. The hydrophobic PLA outer layers on both sides are loaded with erythromycin and puerarin, which possess antibacterial properties. This laminate film dressing demonstrated excellent mechanical properties, high water vapor permeability, and promoted wound healing in a diabetic animal model.^194^^,^^195^

Surgical meshes are primarily used for tissue repair and reconstruction surgeries, such as hernia repair and soft tissue repair. The biocompatibility and biodegradability of PLA material make it an ideal choice for surgical meshes.^196^ Abdominal wall hernias are conditions where visceral organs protrude due to weakened or lost continuity of the fascia or muscle, typically requiring surgical intervention.^197^ In most hernia surgeries, mesh implants are commonly used for reinforcement. PLA materials are often blended with other polymers to enhance performance, improve thermal stability, and increase cell adhesion, as well as to add antimicrobial coatings.^198^^,^^199^ PLA meshes provide a temporary support structure that helps repair abdominal wall defects while allowing new tissue to grow. During the healing process, the PLA mesh gradually degrades and is eventually replaced by newly formed tissue.^200^^,^^201^ According to research, a novel 3D-printed PLA mesh combined with acellular dermal matrix composite material was used to repair abdominal wall defects in rats. This composite scaffold can effectively reduce surrounding inflammation and significantly promote the repair of abdominal wall defects.^202^

### Biodegradable polymeric stents and balloons

Inserting cardiovascular stents is a crucial therapeutic approach for addressing coronary artery diseases. Compared with bare-metal stents, the development of bio-resorbable stents provides new potentials.^203^ The degradation of bio-resorbable stents left no foreign objects in the blood vessel for an extended period, and thus the risks of late and very late stent thrombosis may be reduced or even eliminated.^204^ The PLA polymer-based stents can effectively intermediate revascularization of coronary artery lesions and demonstrate relatively low rates of major adverse cardiac events in early follow-up.^173^^,^^205^ The Igaki-Tamai stent (Kyoto Medical Planning Co., Ltd., Kyoto, Japan) made of PLA is the pioneering bio-resorbable stent employed in human patients, which undergoes self-expansion at body temperature until it reaches equilibrium with vessel wall dilation and resistance.^206^ Biodegradable stents present an appealing alternative to self-expanding stents for managing biliary, coronary, and various duct-related ailments,^207^ where other chemical components can be incorporated into PLA to tailor the characteristics, e.g., ranging from soft and elastic materials to rigid and high-strength materials.^208^ There are also reports about the implantable, biodegradable and inflatable balloons made by polymers of PLA,^209^ where the balloons were developed as a sub-acromial spacer in the treatment of extensive irreparable rotator cuff tears.^210^

### Antimicrobial materials and surgical instruments

Antimicrobial materials are crucial in the medical field, particularly in surgical environments, to prevent infections and promote patient recovery.^196^ Several additives, including natural compounds, peptides, enzymes, metals, chelating agents, and antibiotics, have been incorporated into PLA polymer matrix to impart antimicrobial activity.^211^^,^^212^^,^^213^^,^^214^^,^^215^

PLA silver ion nanoparticles are synthesized by introducing silver ions into the PLA matrix. These nanoparticles combine the biocompatibility of PLA with the broad-spectrum antimicrobial properties of silver ions, resulting in significant antimicrobial efficacy.^216^ Silver ions can disrupt bacterial cell membranes, interfere with bacterial DNA replication, and inhibit protein synthesis, effectively hindering bacterial growth. As a carrier, PLA allows for the sustained release of silver ions, extending the antimicrobial action, and degrades gradually within the body, reducing the side effects in long term.^217^^,^^218^ Recent studies have reported a new antimicrobial surgical retractor manufactured using 3D printing technology with PLA materials, and a thin layer of silver ion PLA nanoparticles is uniformly deposited and fixed on the surface via sonochemical thin-film deposition technology. This retractor exhibits excellent antimicrobial properties and costs only one-tenth of the stainless-steel retractor.^216^

Overall, surgical tools such as scalpels, sutures, and needles can be coated or embedded with PLA-metal nanoparticles to create a durable antimicrobial layer that protects wounds from bacterial invasion.^219^ Additionally, these nanoparticles can be used to produce antimicrobial dressings, catheters, and implants, providing extensive antimicrobial protection.^220^

## Summary and prospects

### Challenges in characteristics of the PLA materials

Controlled biodegradability is considered the core value of PLA materials, but matching the degradation rate precisely with the clinical treatment cycle is still a challenge.^221^ The degradation of PLA scaffolds needs to establish a give-and-take relationship with the regeneration of human tissues, and the drugs released from PLA nanoparticles also need to meet the pharmacokinetic demand of targeted tissue. Therefore, research efforts have been made to investigate the degradation rate of PLA materials in different microenvironments of the human body, e.g., tumor microenvironments, skeletal muscle tissues, blood vessels, etc.^222^ Additionally, it is worth considering the local inflammatory effects caused by the lactic acid produced during PLA degradation. PLA is generally considered nontoxic and biocompatible, but when it degrades rapidly and the local tissue circulation is restricted, the accumulation of lactic acid may cause a decrease in pH, possibly leading to inflammation and tissue irritation, although the syndrome is usually limited and manageable.^223^^,^^224^ Therefore, PLA material design needs to consider controlling the degradation rate to prevent excessive lactic acid buildup.

The tensile strength of PLA is moderate among bioplastics, sufficient for static load applications, and PLA has a high compressive strength, allowing it to withstand substantial compression loads. It also possesses relatively high stiffness and modulus, providing good hardness and stability.^10^^,^^225^ However, PLA has limitations in terms of brittleness and ductility, making it more prone to fracture under impact or tensile stress.^226^ Additionally, its heat resistance is insufficient, with a glass transition temperature typically ranging from 60°C to 65°C, which may lead to softening and deformation *in vivo.*^227^ When applied in the human body, the mechanical strength of PLA can become unstable under prolonged exposure to body temperature and humidity.^228^ In tissue regeneration scaffolds, the porous and loose structure of PLA is beneficial for cell adhesion and growth, but it also reduces mechanical strength, compromising the long-term stability of the scaffold.^229^ Therefore, new modification strategies are needed to expand the application range of PLA materials. Enhancing the mechanical performance of PLA can be achieved through copolymerization, blending, the incorporation of additives or fillers, and optimization of processing techniques.^10^^,^^225^^,^^230^^,^^231^

The hydrophobicity of PLA material is a disadvantage for medical applications by impairing cell adhesion.^232^ Surface modification methods such as plasma treatment,^233^ chemical coating^234^ or UV light-induced free radical reactions can be used to increase the hydrophilicity of PLA,^235^ and then enhance cell adhesion and extracellular mineralization.^236^ Additionally, blending PLA with other hydrophilic materials can increase the hydrophilicity of the composite.^237^ However, uneven dispersion of the hydrophilic materials within the PLA matrix may happen during blending, leading to phase separation.^238^ For example, when blending natural fibers with PLA, insufficient interfacial adhesion between the fibers and the PLA matrix can lead to uneven fiber distribution and reduced mechanical properties.^167^ Using chemical coupling agents or compatibilizers, along with optimizing the blending process, can improve the interface bonding between PLA and hydrophilic materials, reduce uneven dispersion, and thereby enhance the overall mechanical properties and uniformity of the composite.^239^ Enhanced hydrophilicity of PLA materials has been shown to improve cell adhesion and proliferation, thereby promoting tissue regeneration and extracellular mineralization in bone repair, and also accelerating wound healing.^236^

### Promoted PLA clinical applications with advanced manufacturing

Currently, 3D printing technology has been widely utilized in the processing of PLA materials, including bone defect implants based on the precise 3D modeling of imaging data, and porous, biologically labeled, multi-phase tissue regenerative scaffolds.^240^^,^^241^^,^^242^ The newly proposed concept 4D printing refers to the additional feature that products printed in 3D can undergo shape and structural changes when stimulated externally.^243^ The combination of PLA materials with 4D printing may find applications in bioresorbable coronary stents.^232^ When placed inside blood vessels, a bioresorbable coronary stent changes its shape in response to temperature variations after coronary revascularization and degrades over time to reduce the occurrence of blood clots.

Another concept to be mentioned is 3D bioprinting, which produces more complex structures to meet the requirements of multi-level and multi-space regeneration engineering.^244^ Currently, PLA has been used in 3D printing to make a cartilage-bone biphasic scaffold for knee joint cartilage repair or a bone-ligament-muscle triphasic scaffold for ligament regeneration.^243^ In the field of skin regeneration engineering, multiphase PLA scaffolds have been proposed to include epidermis, dermis, blood vessels, and nerves, along with PLA hydrogels or nanoparticles loaded with antimicrobial drugs and growth factors. Furthermore, 3D bioprinting may integrate stem cell materials into printing inks, which facilitates complete organ regeneration on PLA scaffolds.

## Conclusions

PLA has established itself as a remarkable and versatile material in the realm of medical applications. Its biocompatibility, biodegradability, controlled degradation, and versatile processing have made it indispensable in various medical fields, ranging from drug delivery systems, fixation devices and implants, tissue regenerative engineering to wound management and beyond. Nevertheless, the PLA material itself still has some drawbacks, which can be further improved in the future through material modification and advanced manufacturing. In summary, the PLA materials have shown a promising future in medical innovation and will contribute significantly to healthcare advancement and patient well-being.

## Acknowledgments

This research is supported by the program CNSB (Carbon-Negative Synthetic Biology for Biomaterial Production from CO2) under the Campus for Research Excellence and Technological Enterprise (CREATE) of National Research Foundation, Prime Minister’s Office, Singapore.

## Author contributions

Conceptualization, P.X. and S.S.; investigation, Z.Y.; writing – original draft, Z.Y.; writing – review and editing, S.S.; visualization, Z.Y.; supervision, P.X. and S.S.; project administration, G.Y. and P.X.

## Declaration of interests

The authors declare no competing interests.
